# Narrow-diameter implants with conical connection for restoring the posterior edentulous region

**DOI:** 10.1186/s40902-016-0077-x

**Published:** 2016-08-05

**Authors:** In-Hee Woo, Ju-Won Kim, So-Young Kang, Young-Hee Kim, Byoung-Eun Yang

**Affiliations:** 1 Division of Oral and Maxillofacial Surgery, Hallym University College of Medicine, Chuncheon, Republic of Korea; 2 Department of Oral and Maxillofacial Implantology, Graduate School of Clinical Dentistry, Hallym University, Chuncheon, Republic of Korea; 3 Statistical Analysis Department, Korea Health and Welfare Information Service, Seoul, Republic of Korea; 4 Division of Oral and Maxillofacial Radiology, Hallym University College of Medicine, Chuncheon, Republic of Korea

**Keywords:** Dental implant platform switching, Conical dental implant-abutment connection, Narrow-diameter implants, Posterior edentulous

## Abstract

**Background:**

The objective of this retrospective study was to show results from platform-switched narrow-diameter implants in the posterior edentulous region, which we followed up for more than 1 year after functional loading.

**Methods:**

Ninety-eight narrow implants were inserted into 66 patients. After healing, fixed implant-supported prostheses were delivered to the patients, and Periotest and radiographic examinations were performed. After the first year of loading, the implant outcome was again evaluated clinically and radiographically using the Periotest analysis. Crestal bone loss and Periotest values (PTVs) were used to evaluate the effect of surgery, prosthesis, implant, and a host-related factor. A general linear model was used to statistically detect variables statistically associated with crestal bone loss and Periotest value.

**Results:**

We followed up on the implants over 1 to 4 years after loading; their survival rate was 100 %, and pronounced differences from PTVs were noted among jaw location, bone quality, and loading period. No difference was detected in bone loss among the variables studied. Bone loss after functional loading was 0.14 ± 0.39 mm. The stability value from the Periotest was −3.29 ± 0.50.

**Conclusions:**

Within the limitations of this study, judicious use of platform-switched narrow implants with a conical connection must be considered an alternative for wide-diameter implants to restore a posterior edentulous region.

## Background

Edentulous alveolar ridges less than 5 mm wide require horizontal augmentation or expansion to position regular implants and produce the necessary bone quantity (at least 1 mm of bone on the buccal and oral side) [1]. Various surgical techniques including ridge splitting, ridge expansion, lateral augmentation, and horizontal distraction osteogenesis are necessary to increase bone availability in the narrow alveolar ridge. However, these procedures can be problematic in terms of cost-effectiveness, surgery time, and healing time. Katranji et al. demonstrated that the average cancellous thickness ranges from 2.86 to 4.54 in the edentulous mandible and maxilla in the molar region, and the average cancellous thickness ranges from 2.12 to 3.11 in the edentulous maxilla and mandible in the premolar region [2]. Approximately, 3.0- to 4.5-mm-diameter implants should be applied to the edentulous area for bicortical stabilization by engaging both buccal and lingual cortical bones for consistency with Katranji’s report. It is recommended that wide diameter and longer length of implants are installed in the posterior region given certain biomechanical aspects. However, a guarantee of long-term success for wide implants is controversial. Shin et al. reported that the survival rate for a regular-diameter implant is higher than that for wide-diameter implants [3]. They noted that this result might be related not only to specific implant design features but also to the relative relationship of the implant to the host bone dimensions [3]. Typically, 3.75-mm diameter implants are considered standard or regular, which is below and above implants with narrow and wide diameters, respectively [4]. Although various reasons are mentioned for diameter selection, no study clearly supports such explanations. In choosing the implant diameter for the posterior region, the most important factors are the emergence profile, the residual bone width, and occlusal force. Occlusal force decreases with age [5], and likely, adequate occlusal force is proportional to the remaining bone quantity, especially in elderly patients with a narrow ridge [6, 7]. Such observations indicate a varied occlusal force that corresponds to age, race, and sex. A retrospective study using 202 narrow-diameter implants reported a 96 % success rate [8]. An additional study that used 30 narrow-diameter single implants and followed up for 3 to 7 years reported that one fixture was fractured [9].

However, the literature is sparse on reports that evaluate narrow implants in the posterior area. The aim of this study was to evaluate the clinical outcome, survival rate, bone loss, and mechanical and prosthetic complications for narrow implants as well as follow up for more than 1 year after functional loading in the posterior edentulous region.

## Methods

**Fig. 1:**
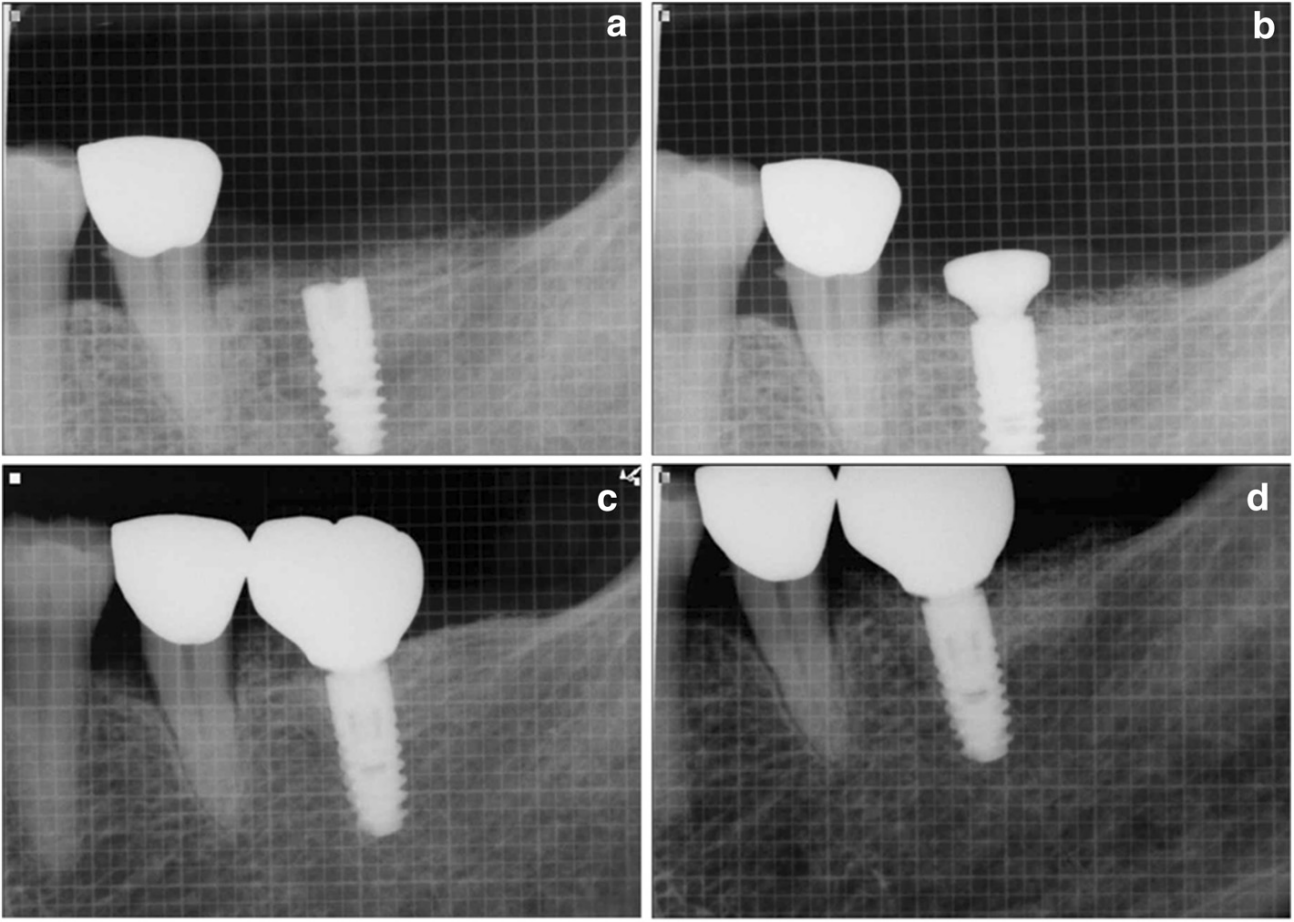
Crestal bone level after implant placement (**a**), 3 months after placement (**b**), after prosthetic restoration (**c**), and after 4-year follow-up (**d**)

This retrospective study was approved by the Ethics Committee at the Hallym University Sacred Heart Hospital (IRB #2013-I007). The patients included herein were consecutively recruited and treated at the Department of Dentistry in the university hospital. From August 2005 to December 2008, 66 patients received a total of 98 Ankylos® implants (Dentsply-Friadent GmbH, Mannheim, Germany) (Fig. 1) with a 3.5-mm diameter. The implants were used in the posterior ridges for complete or partial edentulous patients with an alveolar bone width smaller than 6 mm. We recorded the age, sex, installation site, bone quality at the installation site, length of implants, and periods from installation to the uncovering surgery. The implant surgeries were conducted by one surgeon, and the prostheses were fabricated by one prosthodontist.

The crowns or bridges were glued onto the abutments using provisional cement (TempBond®; Kerr Co.,USA). A radiographic examination was performed using a paralleling technique with a device (Dentsply Rinn, Elgin, IL, USA) and a digital imaging software system (PiViewSTAR® (INFINITT, Korea)). Intraoral digital radiographs were generated at the prosthesis delivery and 6-month intervals after loading. The crestal bone levels were measured using the vertical distance from a fixture reference point from the bone level. The reference point was the most coronal point to the vertical portion of the fixture [10]. The distal and mesial sides of each implant were measured, and a mean value per implant was then calculated. The geometry of the implant was used to assess the distortion of the images. Crestal bone levels that were coincident with the top of the fixture or coronal to it were given the value 0. An electronic mobility-testing device (Periotest; Siemens AG, Bensheim, Germany) was used for the measurements in accordance with the manufacturer’s recommendations on the final follow-up date after final prosthesis removal. The measurements were performed by a single rater, who was blind to the treatment condition. The electronic mobility-testing device provided reproducible data related for the bone-implant complex [11]. The Periotest values (PTVs) ranged from −8 to +50. A range from −8 to +9 corresponded to zero on the Miller index [12]. Statistical significance was *p* < 0.05. Bone loss and PTVs were classified according to the variables. A general linear model was used to discern variables associated with bone loss and PTVs. Statistical data analyses were conducted using SAS version 9.1 (SAS, Cary, NC) and SPSS version 12.0 (SPSS, Chicago, IL, USA).

## Results

The patients’ ages ranged from 19 to 76 years (37 men and 29 women), and the mean age during surgery was 51.4 ± 14.1 years. Bone loss after functional loading was 0.14 ± 0.39 mm. The stability value using the Periotest was −3.29 ± 0.50. Table 1 shows the distribution for the implant positions. Implants 11 mm long were used most frequently. Table 2 shows the distribution for the implant positions. Bone quality was classified in accordance with Professor Nentwig’s standards [13]. Hard-type bone was most commonly observed. The PTV was smallest for implant in hard-type bone (*p* < 0.001). The PTV was smaller for implants in the mandible compared with the maxilla (*p* < 0.001). The PTV for the over 3-year loading group was smaller than that for the over 1-year or 2-year groups (*p* = 0.014). Thirty single implants were used, and the 68 implants were splinted to other implants. Twenty-eight implants were splinted to other narrow implant, and the remaining 40 implants were splinted to wider implants. The remaining variables were not statistically significant (Table 3). The time between insertion and the second surgery was 17.93 ± 10.75 weeks, and a one-stage approach was used in two cases. The time between the insertion and prosthetic treatment was 24.67 ± 9.68 weeks on average. The time between the prosthetic delivery and final follow-up was 37.45 ± 12.80 months. Stability increased with time. There was no significant difference between the sexes in PTV (Table 4). Table 4 shows the association between the variables and PTV using a regression model. There was no statistical association between the variables and bone loss (Table 5). No failure was detected throughout the follow-up dates, except for one screw that was loose in a single implant on the left first molar area 6 months after functional loading, and one prosthesis was detached on the left second molar area from cement loss in a bruxing patient 3 months after functional loading. Figure 1 illustrates the clinical situation in the study before and after 4 years of treatment with narrow implants.

**Table 1:** Length distribution

Length (mm)	Number
8.0	10
9.5	29
11.0	59

**Table 2:** Distribution of implant positions

									Sum
Number of implants		1	7	12	7	8	5	2	42
Tooth position	17	16	15	14	24	25	26	27	
	47	46	45	44	34	35	36	37	
Number of implants	3	12	9	6	8	8	8	2	56

**Table 3:** Comparison of bone loss and PTV by variable

	Number of implants	Bone loss mean (SD)	*p* value	PTV mean (SD)	*p* value
Bone quality			0.967		<0.001
Soft	13	0.15 mm (0.376)		−2.323 (0.208)^a^	
Normal	37	0.15 mm (0.423)		−3.273 (0.266)^b^	
Hard	48	0.13 mm (0.375)		−3.562 (0.335)^c^	
Location			0.402		<0.001
Maxilla	42	0.18 mm (0.439)		−2.979 (0.514)	
Mandible	56	0.11 mm (0.351)		−3.522 (0.335)	
Loading period			0.715		0.014
Over 1 year	22	0.08 mm (0.282)		−3.125 (0.518)^a^	
Over 2 years	19	0.21 mm (0.535)		−3.095 (0.424)^a^	
Over 3 years	34	0.16 mm (0.439)		−3.409 (0.452)^b^	
Over 4 years	23	0.11 mm (0.259)		−3.465 (0.508)^b^	
Splinting status			0.407		0.968
Yes	68	0.16 mm (0.418)		−3.295 (0.519)	
No	30	0.08 mm (0.245)		−3.290 (0.406)	
Reason for tooth loss			0.314		0.559
Periodontitis	89	0.13 mm (0.384)		−3.285 (0.514)	
Caries	9	0.29 mm (0.488)		−3.400 (0.224)	

*T* test or general linear model

*SD* standard deviation, *PTV* Periotest value

^a, b, c^Results from multiple comparisons using the general linear model

**Table 4:** Association between variables and PTV using the regression model

Variable	Univariate model				Multivariate model			
	Parameter	Standard	*t* value	Pr > |*t*|	Parameter	Standard	*t* value	Pr > |*t*|
	Estimate	Error			Estimate	Error		
Bone quality	−0.528	0.047	−11.213	0.000	−0.669	0.084	−7.969	0.000
Jaw location	−0.544	0.085	−6.401	0.000	0.089	0.158	0.565	0.574
Loading period	−0.133	0.044	−3.046	0.003	−0.085	0.030	−2.835	0.006
Splinting status	−0.005	0.125	−0.040	0.968	0.036	0.087	0.417	0.678
Reason for tooth loss	−0.094	0.156	−0.600	0.550	0.078	0.140	0.554	0.581
Sex	−0.101	0.100	−1.016	0.312	−0.080	0.073	−1.106	0.272
Age	−0.001	0.004	−0.348	0.729	0.002	0.003	0.679	0.499
Implant length	0.078	0.049	1.601	0.113	−0.012	0.036	−0.348	0.729

In the regression model and multivariate model, dependent variables were corrected for multiple comparisons

**Table 5:** Association between variables and bone loss using regression model

Variable	Univariate model				Multivariate model			
	Parameter	Standard	*t* value	Pr > |*t*|	Parameter	Standard	*t* value	Pr > |*t*|
	Estimate	Error			Estimate	Error		
Bone quality	0.014	0.056	0.247	0.805	−0.027	0.102	−0.269	0.789
Jaw location	0.067	0.079	0.841	0.402	0.432	0.192	2.257	0.026
Loading period	−0.006	0.036	−0.155	0.877	−0.01	0.036	−0.278	0.782
Splinting status	−0.081	0.098	−0.833	0.407	−0.172	0.106	−1.631	0.106
Reason for tooth loss	−0.073	0.123	−0.593	0.555	0.017	0.17	0.1	0.921
Sex	0.077	0.078	0.987	0.326	0.082	0.088	0.933	0.353
Age	0.005	0.003	1.676	0.097	0.005	0.004	1.42	0.159
Implant length	0.009	0.039	0.244	0.808	0.004	0.043	0.089	0.93

## Discussion

In this study, no implants were lost, and two implants had biomechanical problems during the follow-up period. There is a risk for restoring a posterior region using narrow-diameter implants due to the high masticatory force in molar area [14]. Fatigue fracture of the narrow implant body from mechanical weakening has been reported [15]. It has been suggested that a 4-mm-wide implant has a 30 % higher fatigue resistance than a 3.75-mm-wide implant [16]. Therefore, many previous studies reported that wide implants provide better biomechanical characteristics [17], but under certain circumstances, it is difficult to use regular or wide implants. When wide implants are installed in narrow ridges, many clinicians must bone graft around the fenestrated implant surfaces. However, it is postulated that peri-implant grafted bone will be resorbed if the grafted bone does not have an optimal osteogenesis period.

Bone augmentation procedures are often necessary to enlarge the bone width and facilitate regular- or wide-implant positioning. Autogenous bone grafts require complex surgical techniques, and additional risks must be considered. Using narrow implants gives an unskilled clinician surgical freedom and is applicable in patients without the bone width required for regular-diameter implant installation. Although no fenestration has been reported around the implants when narrow implants are used, bone grafts can be necessary to clear a food bolus. In this study, bone grafts over the alveolar bone using slow-resorbing material such as Bio-Oss® (Geistlich, Wolhusen, Switzerland) were used to restore the optimal alveolar bone width for the buccinator mechanism [18].

Using narrow implants reduces the chance of bone dehiscence or fenestration during a flapless surgery. It also prevents lingual dehiscence in the mandibular second molar area during preparation. Given the decreased width of the drills and implants, osteotomy preparation implies less risk of overheating the bone.

**Fig. 2:**
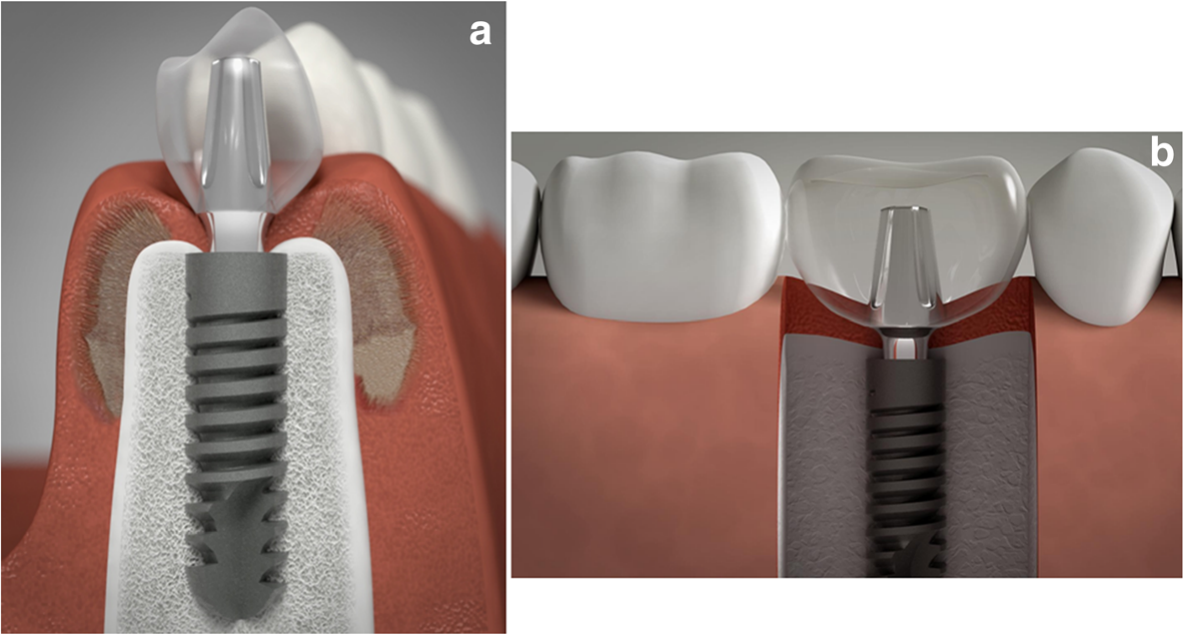
Illustration of an Ankylos® dental implant with a 3.5-mm diameter which was installed in mandibular premolar (**a**) and molar area (**b**). The integrated platform-switching design provides more space for soft tissue retention. The illustration was a courtesy of the Graphic Designer, Ernesto Pacheco (pachecojake@mac.com)

Implants positioned too close together can reduce the height of the inter-implant bone crest. It has been suggested that a distance less than 3 mm between two adjacent implants increases bone loss [19]. Narrow implants enable clinicians to easily generate this distance easily. The greatest challenge in replacing missing teeth with implant restoration is for thin gingival biotype cases. Preserving the bone architecture is paramount to a successful final outcome and the peri-implant soft tissue stability. Clinicians want to create an effective barrier to protect the underlying bone from intraoral microorganisms and by-products [20]. Presumably, Ankylos® system provides more space for the soft tissue retention because it has a narrow connection size that produces greater gingival thickness using the platform switch. Tight and stable soft tissue integration during implant restoration facilitates long-term success (Fig. 2).

The fixture-abutment connection type is also important for implant longevity. Quek et al. reported that narrow-diameter implants are more easily broken than wider implants because they have a narrow platform diameter [21]. However, different results are expected from implants with an internal connection. Certain studies have shown that the biomechanical stability of internal conical connection implants is better than in butt-joint implants [22, 23]. Herein, we used implants with a conical connection, and the force on implants with a conical connection was not focused on a screw but a connection.

In the previous study, the effect of the joint design on the fatigue strength and failure mode in the conical connection system was significantly better for the butt-joint system [22]. Therefore, it would be difficult to apply Quek’s result [21] to the system used herein. In an article published by Zipprich et al., 10 implant systems that used either conical or flat-to-flat connections were compared relative to their dynamic lateral load responses under simulated clinical conditions. The clearance-fit systems produced micromovements, whereas the systems with a conical fit (Astratech® and Ankylos®) generated no movement at all [24]. There will be no micromovement during functional load, and fewer loads on the abutment screws produce few if any screw loosening problems.

One of the pitfalls in using narrow implants is the risk of fracture to the fixture or abutment. The thickness of the fixture titanium wall is important. Where the fixture titanium is too thin around the abutment, the tendency is to lose bone upon loading. Therefore, it is important to secure sufficient fixture titanium around the abutment. A previous study showed that reinforcing the neck region is necessary in reduced-diameter Straumann® tissue level implants [25]. Quaresma et al. reported that a conical-connection implant produces lower stress on the alveolar bone and prosthesis and greater stress on the neck portion of the abutment-prosthesis complex [26]. It has a weak point at the neck portion of the abutment, especially when it is used in the posterior region. Herein, we observed no abutment fracture cases. We presumed that narrow fixtures were generally used in the narrow width of the bone because patients with a narrow bone width may have a weaker occlusal force than patients with normal- or wide-width bones [6]. Interestingly, the Ankylos® system design generates the same abutment for 3.5-mm-, 4.5-mm-, 5.5-mm-, and 7-mm-diameter implant fixtures. Thus, the crown margin position is only determined by the abutment, and after healing, several millimeters of play are available to define the final emergence profile; further, the same connection size between fixture and abutment can be used for all regions in the mouth. If the bone width is wide enough for installation of a wide implant, there is no reason not to use wider implants. However, it should also be noted that sufficient bone housing around the implants may be more important than implant diameter. The premise should be biomechanical stability. In Asian patients, who usually have narrow ridges and thin gingival biotype, using narrow implants enables bicortical installation in the posterior region. However, when treating patients with severe bruxism, heavy masticatory forces and oral habits, a narrow implant is not advised. We observed one case of cement loss in a bruxing patient. The loading history of the implant and the time required for the functional adaptation of the bone to implants may be more important than the implant itself. Development of better biomechanical properties in implants will facilitate narrow implant use in the posterior region. No statistically significant association between the variables and bone loss were detected herein given the combination of the factors described above, such as platform switching and conical connection. A single factor does not produce implant treatment success. Streckbein et al. also reported that low levels of bone strain are observed where a platform switch compensates for a small cone angle in the Ankylos® system [27]. For the PTV value, the groups with a loading period over 3 years is smaller than the group with a loading period of over 1 or 2 years, which is likely related to bone remodeling completion.

## Conclusions

Within the limitations of this study, the prognosis for narrow implants with a conical connection in the posterior region is comparable to wider implants. Thus, narrow implants can likely be used successfully. However, further research is necessary to determine the long-term success of narrow implants in the posterior edentulous region.
